# Mediterranean Diet, Inflammation, and Neurovulnerability: Towards Personalized Nutritional Strategies

**DOI:** 10.3390/nu17142269

**Published:** 2025-07-09

**Authors:** Paola Gualtieri, Antonino De Lorenzo, Laura Di Renzo

**Affiliations:** Section of Clinical Nutrition and Nutrigenomics, Department of Biomedicine and Prevention, Tor Vergata University of Rome, Via Montpellier 1, 00133 Rome, Italy; delorenzo@uniroma2.it (A.D.L.); laura.di.renzo@uniroma2.it (L.D.R.)

The growing focus on the connection between eating behavior, body composition, and neuro-metabolic regulation reflects an urgent need to understand how nutrition impacts not only energy metabolism but also brain health. The Special Issue “Eating Behaviors, Body Composition and Neuro Vulnerability in Energy Metabolism Regulation” gathers publications addressing these topics through a multidimensional lens, emphasizing the necessity of an integrated and personalized approach.

Currently, there is a noticeable erosion of the Mediterranean Diet, which is well established as the most effective nutritional pattern to counteract chronic degenerative diseases [1,2,3].

Childhood obesity has been identified as a significant and widely recognized predictor of various cardiovascular risk factors in adulthood [4,5,6]. Numerous studies demonstrate that strong adherence to the Mediterranean Diet from early childhood is associated with a reduced likelihood of developing overweight and obesity in the short term during later childhood [7,8]. Although longitudinal studies investigating the effects of diet from childhood to adulthood are still lacking, following a Mediterranean Diet during adulthood over a 9-year period has been shown to confer long-term benefits on cardiovascular health [7]. Moreover, adherence to the Mediterranean Diet emerges as a key factor across different age groups. The Lookup 7+ project revealed that lower adherence to the Mediterranean pattern is associated with increased adiposity in elderly populations [9]. These findings support the idea that this dietary model promotes favorable cardiometabolic health, thereby reducing adipose tissue accumulation and future metabolic risk, and suggest that dietary quality from a young age has enduring effects on metabolic health [8,10,11].

In this context, combining school-based and family-oriented programs that include nutritional education and adequate physical activity has been shown to be effective in preventing childhood obesity, thus emphasizing the importance of early interventions in shaping healthy eating behaviors and maintaining healthy body composition in children [12].

Such preventive strategies are essential, as neurovulnerability in energy metabolism regulation arises from a complex interplay of genetic, molecular, and environmental factors, including neuro-endocrine dysfunction, neuroinflammation, and oxidative stress. These factors impair brain circuits responsible for appetite control and energy expenditure, favoring the development of metabolic disorders such as obesity and insulin resistance [13]. Promoting healthy lifestyle habits from a young age may help to strengthen the central nervous system’s capacity to regulate energy homeostasis and reduce long-term metabolic risk.

Within this framework, psychiatric disorders, such as anorexia nervosa, are also implicated, where alterations in tryptophan metabolism via the kynurenine pathway appear to play a central role in the link between nutritional status, inflammation, and brain function [14]. These data strengthen the hypothesis of profound interdependence between nutrition, inflammation, and mental health.

In this scenario, it becomes essential to consider not only nutritional aspects but also neuro-metabolic health when defining prevention and treatment strategies. Personalized interventions that simultaneously target dietary balance, inflammation modulation, and neural function improvement could represent an effective pathway to counteract the progression of chronic metabolic diseases.

From this perspective, dietary personalization extends beyond macronutrient selection to include the integration of bioactive compounds and the modulation of inflammatory and oxidative processes, which are particularly relevant in specific clinical conditions [15].

The importance of nutritional personalization is highlighted by a pilot study on lipedema treatment, where a modified Mediterranean ketogenic diet combined with carboxytherapy led to clinical improvements and enhanced body composition [16]. Ketogenic diets are known for their effectiveness in promoting rapid weight loss by reducing carbohydrate intake and modulating energy metabolism. However, in chronic inflammatory conditions such as lipedema, a dietary approach incorporating anti-inflammatory components is more appropriate. For this reason, personalizing a ketogenic diet with a Mediterranean profile, integrating the beneficial effects of foods rich in bioactive compounds such as polyphenols, flavonoids, vitamins, and minerals, represents a promising strategy to modulate oxidative stress, reduce systemic inflammation, and improve endothelial function, thereby promoting more effective clinical and compositional outcomes [16].

In summary, the works collected in this Special Issue underscore the central role of nutrition in energy regulation and neuro-metabolic health. They reinforce the idea that adult metabolic trajectories are shaped during early life through dietary education and diet quality, even though the Mediterranean Diet is surprisingly uncommon among children living in the Mediterranean region itself [17] and should therefore be prioritized as a preventive policy strategy.

Furthermore, the findings support the efficacy of integrated and targeted interventions for complex pathological conditions. Evidence suggests that personalized nutritional approaches, tailored to age, pathophysiological conditions, and sociocultural contexts, represent a concrete pathway to promote well-being and prevention. The next challenge in nutrition research will be to combine clinical efficacy, sustainability, and accessibility. This Special Issue contributes to defining research directions and strategies that support stable metabolic function and promote long-term health through comprehensive and personalized approaches.
